# Genome-wide identification of cancer-specific alternative splicing in circRNA

**DOI:** 10.1186/s12943-019-0996-0

**Published:** 2019-03-08

**Authors:** Jing Feng, Ke Chen, Xin Dong, Xiaolong Xu, Yuxuan Jin, Xinyang Zhang, Wenbo Chen, Yujing Han, Lin Shao, Yang Gao, Chunjiang He

**Affiliations:** 1 School of Computer Science, Wuhan University, Wuhan, 430072 Hubei China; 2 Department of Urology, Tongji Hospital, Tongji Medical College, Huazhong University of Science and Technology, Wuhan, 430030 China; 3 School of Basic Medical Sciences, Wuhan University, Wuhan, 430071 Hubei China; 4 Hubei Province Key Laboratory of Allergy and Immunology, Wuhan, 430071 Hubei China; 5 Hubei Provincial Key Laboratory of Developmentally Originated Disease, Wuhan, 430071 Hubei China

**Keywords:** Circular RNAs, circRNAs, Alternative splicing, Cancer-specific, ccRCC

## Abstract

Circular RNA (circRNA) is a group of RNA families generated by RNA circularization, which was discovered ubiquitously across different cancers. However, the internal structure of circRNA is difficult to determine due to alternative splicing that occurs in its exons and introns. Furthermore, cancer-specific alternative splicing of circRNA is less likely to be identified. Here, we proposed a de novo algorithm, CircSplice, that could identify internal alternative splicing in circRNA and compare differential circRNA splicing events between different conditions (http://gb.whu.edu.cn/CircSplice or https://github.com/GeneFeng/CircSplice). By applying CircSplice in clear cell renal cell carcinoma and bladder cancer, we detected 4498 and 2977 circRNA alternative splicing (circ-AS) events in the two datasets respectively and confirmed the expression of circ-AS events by RT-PCR. We further inspected the distributions and patterns of circ-AS in cancer and adjacent normal tissues. To further understand the potential functions of cancer-specific circ-AS, we classified those events into tumor suppressors and oncogenes and performed pathway enrichment analysis. This study is the first comprehensive view of cancer-specific circRNA alternative splicing, which could contribute significantly to regulation and functional research of circRNAs in cancers.

**Electronic supplementary material:**

The online version of this article (10.1186/s12943-019-0996-0) contains supplementary material, which is available to authorized users.

## Main text

Circular RNA (circRNA) is a type of RNA that has been ubiquitously discovered in many species by high-throughput sequencing in recent years [1]. Previous studies have reported functions of a number of circRNAs, including acting as an miRNA sponge in transcription [2], acting as an RBP sponge [3] and directly regulating the transcription process [4]. Although a number of circRNAs were functionally characterized in human diseases and cancers [5], the internal structure of circRNA remains ambiguous due to potential alternative splicing, which leads to disadvantages in functional research of circRNAs.

Several databases [6, 7] and tools [1, 8, 9] have been developed to identify circRNAs using high-throughput RNA sequencing data. However, few tools were focused on the internal structures of circRNA except CIRI-full [10], which is more suitable for longer RNA-Seq reads (> 250 or 300 bp). Another algorithms CIRI-AS [8] was designed for most of current available RNA-Seq data (50~150 bp), but CIRI-AS focused on independent sample and did not provide the comparison function between samples. Here, we developed a de novo algorithm, CircSplice (http://gb.whu.edu.cn/CircSplice or https://github.com/GeneFeng/CircSplice), which performs stringent pipeline to compare the circ-AS in different conditions and identify the cancer- or sample-specific circ-AS events. We applied this algorithm in clear cell renal cell carcinoma (ccRCC) and bladder cancer and then characterized the patterns of cancer-specific circ-AS. The potential functions of these AS events were also inspected. Our results indicate different patterns and potential functions of cancer-specific circ-AS, which could contribute significantly to regulation and functional research of various cancer-specific circRNA isoforms.

### De novo algorithm to detect cancer-specific alternative splicing in circRNAs

To explore the potential variable internal structures in circRNA, we developed a de novo algorithm named CircSplice, which can identify alternative splicing events in circRNA, also called circ-AS. CircSplice is a Perl script that detects potential circRNA first by back-splicing events. Then, alternative splicing events within back-splicing reads and paired-end reads are identified. Splice sites GT-AG and CT-AC are required to support back-splicing and alternative splicing junctions. Four circ-AS events were identified in CircSplice: skipping exon (SE), retained intron (RI), alternative 5′ splice site (A5SS) and alternative 3′ splice site (A3SS). After the circ-AS events were identified in different samples, for example in cancer and normal tissues, the circ-AS only existing or expressed either in cancer or normal were classified and identified as cancer- or normal-specific. CircSplice is highly efficient that it takes about 10 min to run one 12G sample on a 2.2GB CPU machine with 128GB of memory. The detailed workflow of CircSplice are described in the (Additional file 1: Figure S1).

### Identification and validation of cancer-specific AS of circRNA

**Fig. 1:**
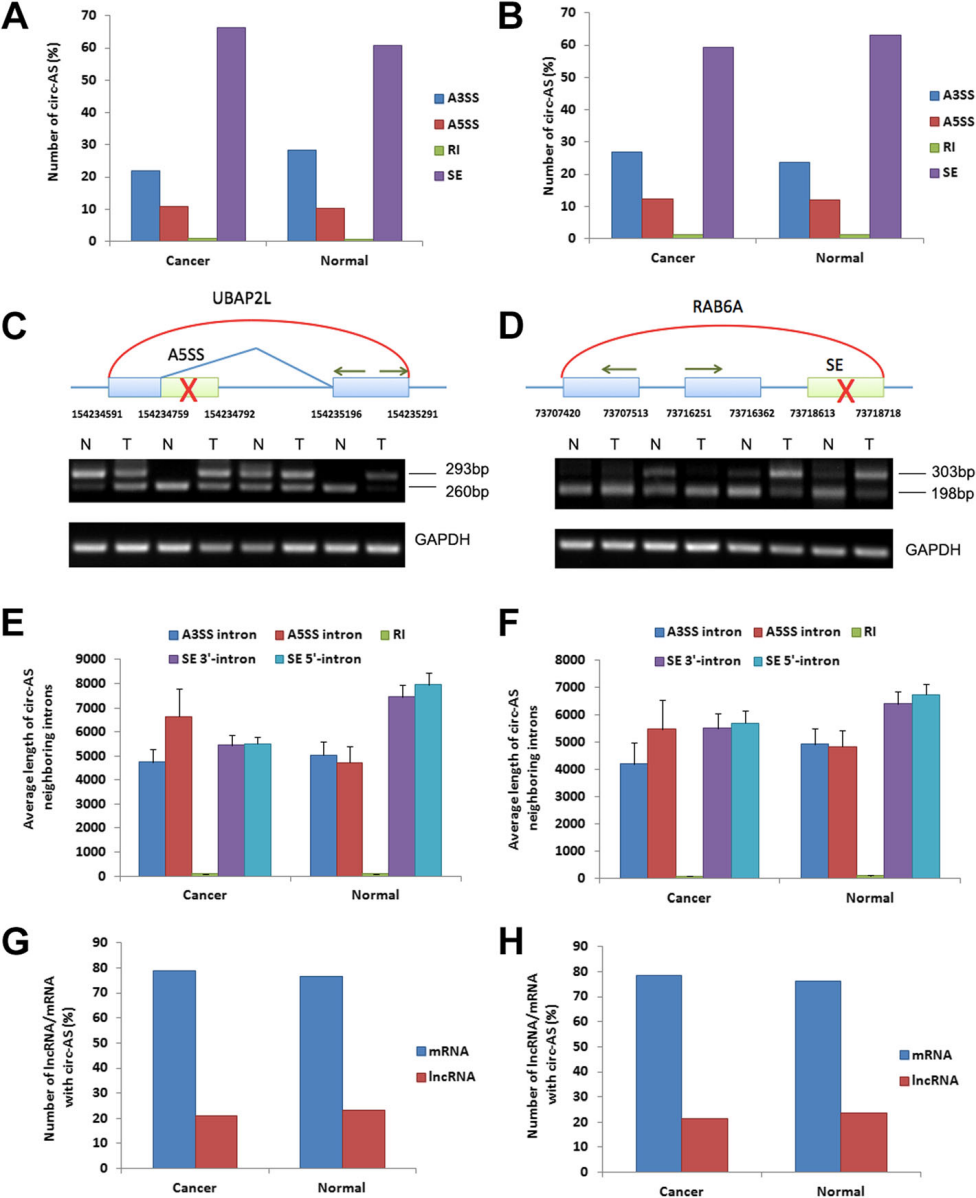
Identification and characterization of circ-AS patterns in cancer and adjacent normal tissues. **a**. The ratio of the four types of circ-AS (SE, RI, A5SS, A3SS) in ccRCC and adjacent normal tissues. **b**. The ratio of the four types of circ-AS in bladder cancer and adjacent normal tissues. **c**. Validation of one A5SS event happened in circ-UBAP2L. Top: The light blue box represents the constitutive exon of circRNA and the light green box represents the alternative exon of circRNA. The arrows represent the position of PCR primers. Bottom: RT-PCR of two circRNA isoforms generated by circ-AS. **d**. Validation of one SE event happened in circ-RAB6A. **e**. The average length of the four types of circ-AS in ccRCC and adjacent normal tissues. **f**. The average length of the four types of circ-AS in bladder cancer and adjacent normal tissues. The error bar represents the Standard Error. **g**. The distribution of circ-AS event in lncRNA and mRNA between ccRCC and adjacent normal tissues. **h**. The distribution of circ-AS in lncRNA and mRNA between bladder cancer and adjacent normal tissues

We performed total RNA sequencing treated with RNase R digestion for 3 paired ccRCC and adjacent normal tissues and applied CircSplice to predict the circRNA and four types of circ-AS events: skipping exon (SE), retained intron (RI), alternative 5′ splice site (A5SS) and alternative 3′ splice site (A3SS). We identified a total of 4498 circ-AS (circ-AS) events in 4826 circRNAs in all 6 samples. Among those, 1799 circ-AS events were only detected in cancer (cancer-specific), 1505 events were only detected in the adjacent normal samples (normal-specific) and 1194 events were detected both in cancer and normal samples (common). In both cancer- and normal-specific circ-AS, there were the largest ratio of SE (66.4 and 60.1%, respectively), more than A3SS (22.0 and 28.3%), A5SS (10.7 and 10.3%) and RI (0.89 and 0.73%) (Fig. 1a). To further confirm the distribution of circ-AS between cancer and normal sample, we analyzed another dataset which performed total RNA sequencing (RNase R treated) for 3 pairs of bladder cancer and adjacent normal tissues and identified total 2977 circ-AS events. Overall, 59.3 and 63.0% of SE were observed in cancer and normal respectively, more than A3SS (27.0 and 23.8%), A5SS (12.4 and 12.0%) and RI (1.31 and 1.11%) (Fig. 1b). Our results indicated different distributions of four AS types in cancer and normal tissues, suggesting the specificity of circ-AS in different conditions.

To validate our prediction results, we selected two AS events and performed RT-PCR in clinical ccRCC and adjacent normal tissues. In circRNA (chr1:154234591–154,235,291) generated from UBAP2L, an A5SS event was detected with differential expression between cancer and adjacent normal tissues. A 33 bp portion of a partial exon was alternatively spliced, causing the circRNA to alter to two isoforms. Considering the heterogeneity of different donor cancer patients, we observed that the two circRNA isoforms were differentially expressed in cancer and adjacent normal tissues (Fig. 1c). Another SE event occurred in circRNA (chr11:73707420–73,718,718) from RAB6A that also caused alternative splicing of a 95 bp exon. The results revealed that the two isoforms of the circRNAs were also differentially expressed between cancer and adjacent normal tissues (Fig. 1d).

### Characterization of cancer-specific AS patterns of circRNAs

As circRNAs were generated with different patterns from regular splicing, the characteristics of circ-AS, was less likely to be inspected. For example, the intron length was involved in the alternative splicing of linear RNAs [11]. Here, to inspect if the intron length was also related to the circ-AS, we detected the distribution of intron length of each type of circ-AS event. The definition of circ-AS intron length was described in the Methods. Our results in ccRCC showed there were longer intron of cancer-specific A5SS (6647 bp) than A3SS (4754 bp). However, in normal-specific events, the average longer intron of circ-AS events was A3SS (5041 bp), longer than A5SS (4734 bp) (Fig. 1e). In bladder cancer, similar results were observed. There were longer A5SS intron (5472 bp) than A3SS intron (4199 bp) in cancer-specific circ-AS, which was reverse in normal-specific results (Fig. 1f). In both two cancers, the normal-specific SE established longer neighboring 5′- and 3′-introns than other two types (A5SS, A3SS and RI), which were not observed in cancer-specific events. The length of RI (retained intron) were the shortest in all circ-AS types. The variance of length of circ-AS neighboring introns established the preference difference of splicing patterns in cancer and normal samples.

Moreover, as circRNAs were discovered both from mRNA and lncRNA [4], to inspect the distribution of circ-AS events in lncRNA or mRNA, we classified those circ-AS into those two types according to their host transcripts. The results showed that 78.8% of cancer-specific circ-AS events were from mRNA, which were approximately four times than that in lncRNA (21.2%) in ccRCC. In contrast, 76.6% of normal-specific events were from mRNA and 23.4% were from lncRNA (Fig. 1g). In bladder cancer, similar results were observed. 78.6 and 21.4% of cancer-specific circ-AS were from mRNA and lncRNA respectively. Comparing to this, 76.2% of normal-specific circ-AS were from mRNA, more than 23.8% of lncRNA circ-AS events (Fig. 1h). This result indicated there were probably more frequent circRNA splicing activity in mRNA than in lncRNA, both in cancer or normal conditions.

### Function exploration of cancer-specific AS of circRNA

**Fig. 2:**
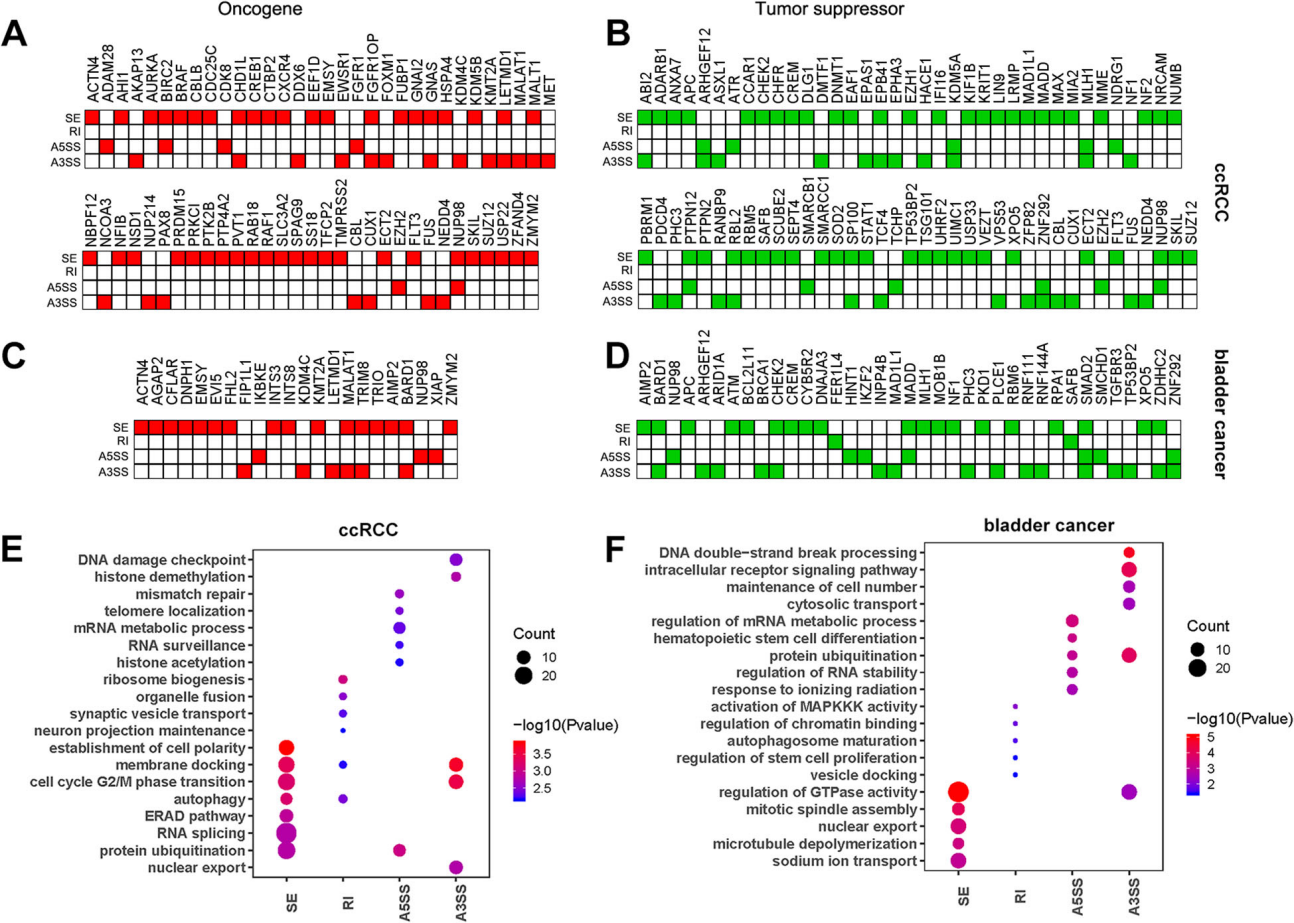
Functional characterization of cancer-specific circ-AS. **a**. The distribution of oncogenes with cancer-specific circ-AS in ccRCC. Red color represents the occurrence of oncogene. **b**. The distribution of TSGs with cancer-specific circ-AS in ccRCC. Green color represents the occurrence of TSG. **c**. The distribution of oncogenes with cancer-specific circ-AS in bladder cancer. **d**. The distribution of TSGs with cancer-specific circ-AS in bladder cancer. **e**. Pathway enrichment of cancer-specific circ-AS in ccRCC. **f**. Pathway enrichment of cancer-specific circ-AS in bladder cancer. GO biological processes are used for this enrichment analysis. The dot color represents the *P-value* which is calculated by clusterProfiler. The size of dot represents the number of genes with circ-AS

Considering the potential involvement of circ-AS in cancer development, we further detected the distribution of cancer-specific circ-AS in oncogenes and TSG. Overall in ccRCC, we identified 63 oncogenes that had cancer-specific circ-AS, including 44 SE, 20 A3SS and 6 A5SS (Fig. 2a). We also identified 75 TSGs that had cancer-specific circ-AS, including 50 SE, 24 A3SS and 11 A5SS (Fig. 2b). In bladder cancer, we also identified 22 oncogenes that had cancer-specific circ-AS, including 16 SE, 6 A3SS and 3 A5SS (Fig. 2c), as well as 37 TSGs that had cancer-specific circ-AS, including 19 SE, 16 A3SS, 7 A5SS and 2 RI (Fig. 2d). The distribution of circ-AS events in oncogenes and TSG suggested circ-AS were potentially involved in cancer processes.

Moreover, to inspect the potential function of the cancer-specific circ-AS events, we performed pathway analysis for the host genes of circ-AS. The results indicated different types of cancer-specific circ-AS established different GO enrichments. For example, the cancer-specific circ-SE in ccRCC was mainly enriched in membrane docking, cell cycle, autophagy, the ERAD pathway, protein ubiquitination and cell polarity. circ-RI mainly enriched on neuron projection, synaptic vesicle transport, organelle fusion and ribosome biogenesis. circ-A5SS were potentially involved in histone acetylation, RNA surveillance, mRNA metabolic, telomere localization and mismatch repair. Lastly, circ-A3SS was mainly observed in nuclear export, histone demethylation, membrane docking and cell cycle (Fig. 2e). The cancer-specific circ-AS in bladder cancer also established differential enrichment of biological processes (Fig. 2f). In summary, these results indicated the functional categories of cancer-specific circ-AS in TSG, oncogenes and various pathways, revealing the involvement of circ-AS in cancers, which can contribute to exploration of circRNA splicing and functions.

### Comparison of CircSplice with CIRI-AS

To indicate the advantages of CircSplice to the previous tool, we applied another published algorithm, CIRI-AS [8], on our ccRCC datasets and compared the results of CIRI-AS and CircSplice. We detected a total 2467 circ-AS events by CIRI-AS, which was less than 4498 events in CircSplice. Then, we compared the results from the two algorithms and observed total 1351 circ-AS events from CIRI-AS were overlapped with CircSplice. Among those, 542 A3SS, 320 A5SS, 18 RI and 471 SE events were overlapped between the two algorithms (Additional file 1: Table S1). Overall, Circsplice detected more circ-AS events except for those events detected by CIRI-AS. CircSplice also performed additional exon correction while detecting circ-AS events, that requires at least one junction of spliced reads is identical to one junction of spliced exons, which could decrease the false positive results significantly. Furthermore, CircSplice provided an integration tool which could compare the circ-AS across samples, which could significantly enhance circRNA studies in different diseases and conditions.

## Conclusions

In summary, we developed a more efficient and stringent tool for detecting circular RNA alternative splicing between cancer and normal conditions and conducted the first comprehensive analysis for distributions and patterns of cancer-specific circRNA alternative splicing, which is important to the function and regulation research of circRNA in cancers. We believe our pipeline and analysis present an important new view for exploring circRNA alternative splicing events that involved in development and disease.

## Additional file

Additional file 1: Materials and methods, supplementary figure and table. (PDF 145 kb)

## Abbreviations

A3SS: Alternative 3′ splicing site
A5SS: Alternative 5′ splicing site
AS: Alternative splicing
ccRCC: Clear cell renal clear cell carcinoma
circ-AS: Circular RNA alternative splicing
GO: Gene oncology
RI: Retained Intron
RT-PCR: Reverse transcription polymerase chain reaction
SE: Skipping exon
