# Supplementary material for: Genome-wide identification of cancer-specific alternative splicing in circRNA
Source: Mol Cancer. 2019 Mar 8;18:35. doi: 10.1186/s12943-019-0996-0 (PMC6408762; doi:10.1186/s12943-019-0996-0)

Supplemental files

Materials and Methods

Workflow of CircSplice

CircSplice is a novel algorithm to identify sample-specific or cancer-specific alternative splicing in circRNA. CircSplice is implemented in Perl and has been tested in Unix and Mac OS X. The overview of CircSplice is established in a web-based resource (http://gb.whu.edu.cn/CircSplice). CircSplice is highly efficient and the workflow is as follows:

a. Sequencing reads were mapped to the genome by STAR, and chimeric reads were collected for the next step.

b. Only one end of paired-end reads mapping to the same chromosome and strand were retained, and the genomic coordinates of each end were extracted according to the CIGAR value.

c. Back-splicing reads were identified, and the other end corresponding to back-splicing reads were required to be located inside the donor and acceptor sites of back-splicing, which ensures the other end was also from circRNA.

d. Splice sites in the back-splicing junction were identified and filtered by GT-AG or CT-AC.

e. Genes were annotated for the back-splicing read.

f. The end with back-splicing and the corresponding paired end were both mapped to annotated gene exons and introns and were identified for 4 types: skipping exon, retained intron, alternative 5’ splice site and alternative 3’ splice site. A 2 bp mismatch was tolerated in the prediction. At least one junction of spliced reads should be identical to one junction of spliced exons.

g. Splice sites in the alternative splicing junction were checked and filtered by GT-AG or CT-AC.

h. The number of reads supporting the alternative splicing events were counted. Normalized read counts were calculated by (the number of reads supporting this event/ total chimeric reads)*10^6.

i. Alternative splicing events in different samples were merged according to genomic coordinates. A 2 bp mismatch was tolerated in the merging.

Sample acquisition

Three pairs of tumor samples and adjacent normal tissues from three ccRCC patients were collected from Tongji Hospital, Tongji Medical College, Huazhong University of Science and Technology. The study was approved by the institutional IRB and consents obtained.

RNA sequencing

Total RNA was extracted from clinical samples, following by treatment with RNase R and rRNA depletion from the total RNA. Then, the RNA was reverse transcribed to cDNA and constructed into a strand-specific library. Illumine hiseq 2500 was performed for the sequencing. All raw data can be accessed in the NCBI GEO database (GSE124453, accession code: uxknyocyrbkfbad). Another dataset including three pairs of bladder cancer and adjacent tissues (NCBI GEO database: GSE97239) were also included in the analysis.

Identification of circ-AS in cancer and adjacent normal tissues

Sequencing reads were filtered by quality control and the cleaned reads were mapped to human hg38 genome. circ-AS events in ccRCC and bladder cancer samples were predicted by Circsplice respectively. Gene annotation including lncRNA and mRNA based on human hg38 genome version by integrating RefSeq [1] were used to annotate the circ-AS. Then, all AS events from cancer and adjacent normal tissues were merged into one file according to genomic coordinates. A 2 bp mismatch was tolerated, according

to previous research [2]. Then, those AS events expressed in cancer or adjacent normal tissues were compared and classified into cancer-specific or normal-specific groups.

Validation of circ-AS by RT-PCR

To validate the expression of circ-AS, we collected cancer and adjacent normal tissues from four ccRCC patients. Total RNA from 5 mouse tissues were prepared by the TRIzol reagent (Invitrogen, Cat 15596026) according to the manufacturer's instructions. All RNA was digested by RNase-free DNase I and purified. RNA (3 μg) was used as a template for RT using the RevertAid First Strand cDNA Synthesis Kit (Thermo Fermentas, Cat K1622). PCR was performed in a 20 μl reaction mixture using 2XPCR Reagent (Tiangen, KT207-1). Primers were designed in internal constitutive exons of circRNA to detect potential alternative isoforms of circRNA. All primers are listed as follows: circ-UBAP2L: Forward: GAACAGAAAGAGGCAGAAGGGG, Reverse: CCAGAAGGCCCTCCACTCTT; circ-RAB6A: Forward: GAGGGAGAGAGGAAAGCCAAA, Reverse: ACACTTGCCTCTTGTCAGCA.

Definition of the length of circ-AS neighboring intron

The length of intron neighboring to each circ-AS was detected for each of the 4 types. For RI, the length of the retained intron were calculated directly. For SE, the length of 5'- and 3'-intron adjacent to the skipping exon were analyzed respectively. For A5SS and A3SS, the length of internal intron resided in the splicing junctions is measured.

Distribution of cancer-specific circ-AS in oncogenes and TSG

The annotation for human oncogenes and TSG was acquired from a public database [3, 4]. Then, the cancer-specific circ-AS events were distributed in oncogenes or TSGs. Heatmaps of the 4 circ-AS types in oncogenes (red) or TSGs (green) were plotted by TMeV4 [5].

Functional enrichment analysis of cancer-specific circ-AS

The host genes of cancer-specific circ-AS were used for pathway analysis. Enrichment analysis of Gene Ontology (GO) biological processes was performed by R package

clusterProfiler [6]. Fisher Exact test was used to calculate the P-value. The top enriched pathways with significant P-values were plotted by TMeV4 [5].

1. Pruitt KD, Tatusova T, Maglott DR. NCBI Reference Sequence (RefSeq): a curated non-redundant sequence database of genomes, transcripts and proteins. Nucleic Acids Res. 2005;33:D501-504.
2. Rybak-Wolf A, Stottmeister C, Glazar P, Jens M, Pino N, Giusti S, Hanan M, Behm M, Bartok O, Ashwal-Fluss R, et al. Circular RNAs in the Mammalian Brain Are Highly Abundant, Conserved, and Dynamically Expressed. Mol Cell. 2015;58:870-885.
3. Liu Y, Sun J, Zhao M. ONGene: A literature-based database for human oncogenes. J Genet Genomics. 2017;44:119-121.
4. Zhao M, Kim P, Mitra R, Zhao J, Zhao Z. TSGene 2.0: an updated literature-based knowledgebase for tumor suppressor genes. Nucleic Acids Res. 2016;44:D1023-1031.
5. Saeed AI, Bhagabati NK, Braisted JC, Liang W, Sharov V, Howe EA, Li J, Thiagarajan M, White JA, Quackenbush J. TM4 microarray software suite. Methods Enzymol. 2006;411:134-193.
6. Yu G, Wang LG, Han Y, He QY. clusterProfiler: an R package for comparing biological themes among gene clusters. OMICS. 2012;16:284-287.

Supplemental Table

Table S1. Number of overlapping circ-AS between CIRI-AS and CircSplice

	# of circ-AS from CIRI-AS	# of overlapped circ-AS	# of circ-AS from CircSplice
A3SS	921	542	1092
A5SS	589	320	488
RI	48	18	35
SE	909	471	2883
Total	2467	1351	4498

Supplemental Figure Legend

Figure S1. The workflow and algorithm of CircSplice. A. The workflow of detection of circ-AS across samples. **B.** Schematic diagram of the identification of alternative splicing in back-splicing reads. Four types (SE, RI, A5SS and A3SS) are defined according to the relative location between reads and the annotated exon/intron. Splice sites in the back-splicing site and the alternative-splicing site are filtered by GT-AG and CT-AC. Light blue box: constitutive exon. Light green box: alternative spliced exon. Pink arc: back-splicing reads. Red line: coordinates of circ-AS in the output.

Supplemental Figure S1

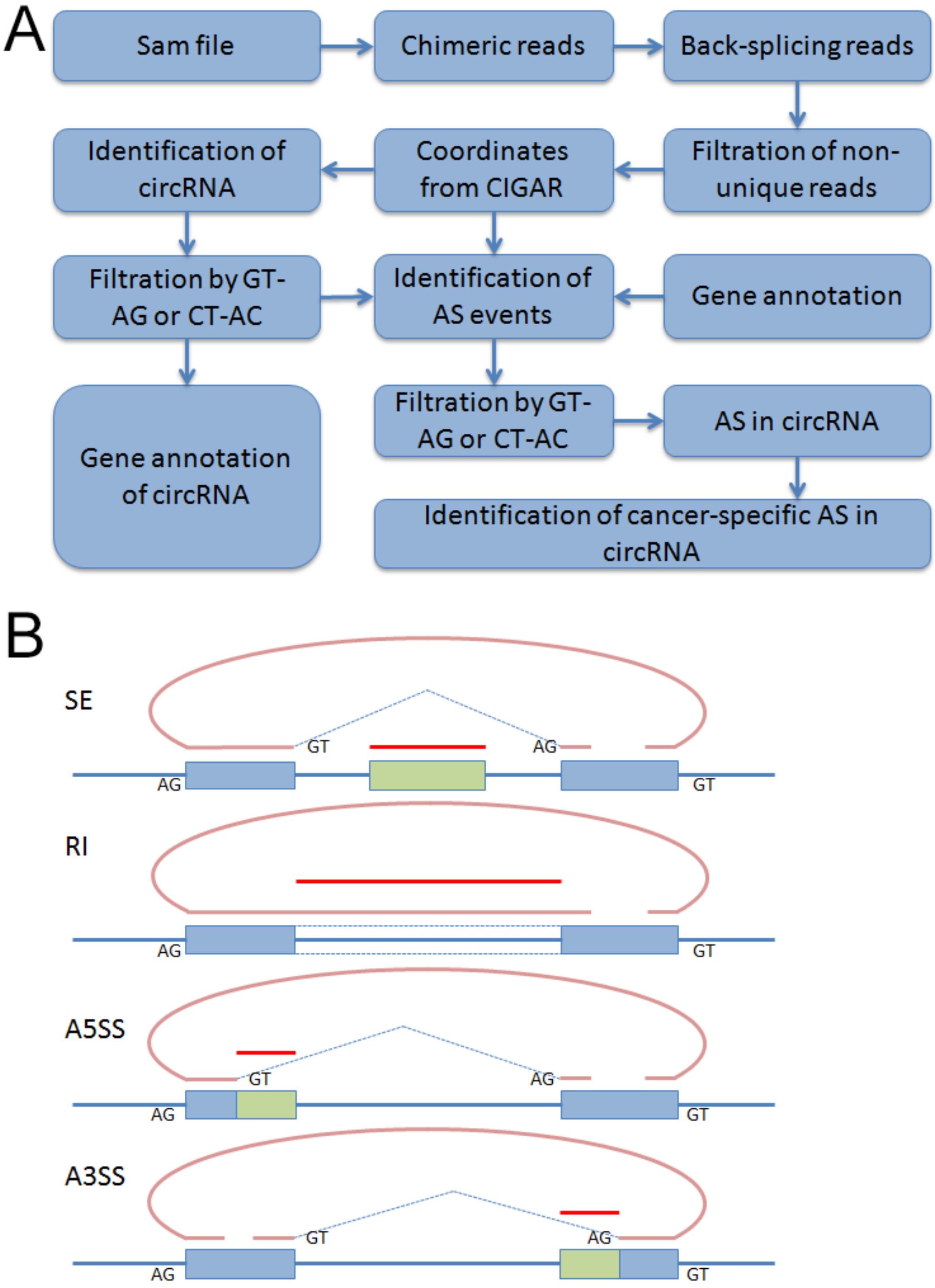

Supplement: Supplementary file 1 — Materials and methods, supplementary figure and table. (PDF 145 kb) [file 12943_2019_996_MOESM1_ESM.pdf]
